# The Attitudes and Practices Regarding COVID-19 among General Practitioners from Croatia and Bosnia and Herzegovina: A Cross-Sectional Study

**DOI:** 10.3390/bs13050352

**Published:** 2023-04-22

**Authors:** Zudi Osmani, Almina Bajrektarevic Kehic, Ivan Miskulin, Lea Dumic, Nika Pavlovic, Jelena Kovacevic, Vedrana Lanc Curdinjakovic, Juraj Dumic, Ivan Vukoja, Maja Miskulin

**Affiliations:** 1 Faculty of Health Studies, University “VITEZ”, 72 270 Travnik, Bosnia and Herzegovina; zudi@bih.net.ba; 2 Institute for Public Health of Central Bosnia Canton, 72 270 Travnik, Bosnia and Herzegovina; 3 Faculty of Medicine Osijek, Josip Juraj Strossmayer University of Osijek, 31 000 Osijek, Croatia; a.bajrektarevic55@gmail.com (A.B.K.); lea.dumic@mefos.hr (L.D.); nika.felicita@gmail.com (N.P.); jelena.kovacevic@mefos.hr (J.K.); vedrana.lanc.curdinjakovic@zzjz-vsz.hr (V.L.C.); ivan.vukoja@mefos.hr (I.V.); 4 Faculty of Dental Medicine and Health Osijek, Josip Juraj Strossmayer University of Osijek, 31 000 Osijek, Croatia; juraj.dumic@gmail.com

**Keywords:** general practitioners, attitude, practice, COVID-19, Bosnia and Herzegovina, Croatia, cross-sectional study, questionnaire

## Abstract

Attitudes toward COVID-19 (coronavirus disease 2019) prevention and control may have influenced general practitioners’ (GPs’) work during the COVID-19 pandemic. The present study aimed to investigate the attitudes and practices of GPs from Croatia and Bosna and Herzegovina regarding COVID-19 prevention and control as well as the factors which may have influenced them. A cross-sectional study using a self-administered, anonymous questionnaire was conducted between February and May of 2022 on 200 Croatian and Bosnian GPs. The study revealed that the attitudes and practices of the surveyed GPs regarding COVID-19 prevention and control were satisfactory. The Croatian GPs reported a larger number of positive attitudes toward COVID-19 prevention and control (*p* = 0.014), while no significant differences in practices were established. Among the Croatian GPs, more positive attitudes toward COVID-19 prevention and control were reported by participants who had finished a formal education on the prevention of infectious diseases and occupational safety (*p* = 0.018), while among the Bosnian GPs, more positive attitudes were reported by older GPs (*p* = 0.007), males (*p* = 0.026), GPs with a longer length of service (*p* = 0.005), GPs who had finished a formal education on the prevention of infectious diseases and occupational safety (*p* < 0.001), GPs who had finished a formal education on adequate hand hygiene (*p* < 0.001), and GPs who had finished a formal education on COVID-19 prevention for GPs (*p* = 0.001). Considering GPs’ practices regarding COVID-19 prevention and control, among the Croatian GPs, more positive practices were reported by older GPs (*p* = 0.008), females (*p* = 0.002), GPs who had a partner (*p* = 0.021), GPs who were specialists in family medicine (*p* = 0.014), GPs with a longer length of service (*p* = 0.007), and GPs who had finished a formal education on the prevention of infectious diseases and occupational safety (*p* = 0.046), while among the Bosnian GPs, no significant correlations were determined. The general practitioners’ sociodemographic and employment characteristics strongly influenced their attitudes and practices regarding the prevention and control of COVID-19. The cultural differences between Croatia and Bosnia and Herzegovina, as well as the organizational specificities of their healthcare systems, probably modified the observed differences in the individual patterns of associations between the outcome and explanatory variables in the surveyed neighboring countries.

## 1. Introduction

Since the beginning of the COVID-19 (coronavirus disease 2019) outbreak, this pandemic has challenged the healthcare systems of all countries around the globe [1]. Croatia and Bosnia and Herzegovina were once parts of the same country, where their healthcare systems functioned within the framework of a centralized, communist system, and after gaining independence both healthcare systems were seriously challenged by devastating wars. The experience of those difficult times has proven that the main strengths of the healthcare systems in both countries were its healthcare workers [2].

Considering the organizational structure of the healthcare systems in Croatia and Bosnia and Herzegovina after their independence, both healthcare systems maintained universal healthcare coverage but developed in different ways. In Croatia, besides the universal healthcare system managed by the State Ministry of Health, there are some limited private healthcare initiatives, mostly attributable to the concession in primary healthcare with several secondary private healthcare institutions, and the country is constantly balancing the development of different healthcare services while considering expenditure and the stabilization of effectiveness and the quality of care [2]. The complexity of Bosnia and Herzegovina’s politics, which comprises three different political systems and entities, namely The Federation of Bosnia and Herzegovina, the Republika Srpska, and one autonomous district, the Brčko District of Bosnia and Herzegovina, is reflected in the organization of the healthcare system, and it can be said that the country now has one of the most complex healthcare systems in Europe [2,3]. Because of the described structure, Bosnia and Herzegovina is one of the few countries in the world that does not have a state ministry of health; instead, it is divided into two main parts: The Federation of Bosnia and Herzegovina, which has ten subordinate cantonal healthcare systems, and the Republika Srpska, which has a centralized healthcare system. In addition, there is also the Brčko District, which is an isolated, standalone unit. To summarize, in Bosnia and Herzegovina there are thirteen official decision-makers in the healthcare system: The Federal Solidarity Fund and ten Cantonal Insurance Funds in the Federation of Bosnia and Herzegovina, the Insurance Fund of the Republika Srpska, and the Insurance Fund of the Brčko District [2]. During the COVID-19 pandemic, all of the aforementioned official decision-makers formed their Headquarters for Emergency Situations and specific approaches to the organization and adjustment of healthcare during this crisis [3].

The COVID-19 pandemic has revealed that all European Union countries have certain weaknesses in their healthcare systems, and one of the most important ones is the shortage of healthcare workers. Following the fact that developed European Union countries are compensating for the described healthcare labor shortages by hiring healthcare workers, especially medical doctors and nurses from the countries of the western Balkans and Croatia, the actual pandemic pointed out one mutual threat to the healthcare systems in Croatia and Bosnia and Herzegovina, and that is a problem with human resources within the systems [4]. The latter presents a serious threat to the resilience of these healthcare systems, bearing in mind that the recent past has shown that the main strengths of the healthcare systems in both countries are their healthcare workers [2].

Since the beginning of the COVID-19 pandemic, the World Health Organization emphasized that primary care is an essential basis for the global response to the COVID-19 pandemic, and that its role in tackling the pandemic is crucial [1,5,6,7,8]. Primary care is the backbone of every healthcare system. It has been proven that a strong primary care system is the basis of an effective and efficient national healthcare system that removes inequalities in healthcare access. The latter is extremely important in situations such as global health crises because it has been shown that in countries with limited access to primary care, the novel coronavirus spread with greater intensity and speed, while countries with strong primary care systems more successfully limited the spread of COVID-19 and prevented the overloading of health institutions at the secondary and tertiary levels of healthcare [9,10]. Primary healthcare workers are usually the first contact for patients presenting with symptoms of COVID-19, acting as some sorts of gatekeepers of the entire healthcare system [6,10,11,12]. Furthermore, only the most severe COVID-19 cases end up in hospitals, while the diagnosis is almost always carried out by general practitioners (GPs), providing treatment and support for the majority of COVID-19 patients who manage their disease at home [1,10,13,14]. Except for the described assignments, GPs play an important health-promoting role during global medical crises such as the COVID-19 pandemic [12,15]. It has been shown that preventative measures are the cornerstone in managing the pandemic, and GPs play a crucial role in disseminating truthful information regarding the mentioned preventative measures and COVID-19 characteristics [12,15,16]. Patients consider GPs as trusted sources for different kinds of advice and medical information, so the potential of their influence on the course of the pandemic in a particular area is enormous [12,17,18]. Furthermore, the communication of GPs with patients regarding the COVID-19 pandemic is Influenced by their knowledge, attitudes, and practices, so it can be said that those variables influence the dynamics of the pandemic in a particular area [19]. Hence, it is obvious that the adequate knowledge, attitudes, and practices of GPs regarding COVID-19 may decrease the risk of infection and impact patient outcomes [9,14,20,21]. The attitudes of frontline healthcare workers such as GPs are therefore of inestimable importance, for it has been shown that there is a strong interconnection between one’s attitudes and practices regarding the same issue [9,21,22,23]. There is a need for a deep understanding and identification of factors that may influence the attitudes and practices of frontline healthcare workers regarding COVID-19 [24]. Therefore, the present study aimed to investigate the attitudes and practices of GPs from Croatia and Bosna and Herzegovina regarding COVID-19 prevention and control as well as the factors that influence established attitudes and practices.

## 2. Materials and Methods

### 2.1. Study Design and Study Participants

According to the latest available official statistics on the number of GPs in Croatia and Bosnia and Herzegovina, there are 2.243 GPs in Croatia [25] and 1.074 GPs in the Federation of Bosnia and Herzegovina [26]. From those 3.317 GPs in both countries, 500 potential participants (15.0%) were chosen by random selection and were sent an anonymous questionnaire via post. Together with the questionnaire, each potential participant received an explanation of the research, an informed consent form, and two envelopes containing the address of the lead investigator in each country. The potential participants were instructed to sign the informed consent form before answering the questionnaire and to put the signed informed consent form in one envelope and the answered questionnaire in the other envelope and then send them to the lead investigator in each country. Following the described process of data collection, the anonymity of participants in the research was ensured, and the personal data of the study participants could not be linked in any way to the answers given in the collected questionnaires. All collected questionnaires that arrived via post were assigned codes, and the data provided within the questionnaires were stored in the database and later analyzed using the assigned codes. The overall response rate was 52.4% (262/500), but the final sample size consisted of 200 participants (100 from each country), since 62 questionnaires were discarded from the statistical analysis because they were incomplete. Accordingly, the study included 4.5% (100/2.243) of GPs who were working in family medicine practice in Croatia and 9.3% (100/1.074) of GPs who were working in family medicine practice in the Federation of Bosnia and Herzegovina, and those samples were considered representative for the population of GPs in the surveyed countries. This cross-sectional study was conducted from February to May 2022. The study was approved by the Ethics Committee of the Faculty of Medicine Osijek, Osijek, Croatia (ethical approval code: 2158-61-07-21-59) and by the Ethics Committee of the University “VITEZ”, Travnik, Bosnia and Herzegovina (ethical approval code: 1810/22).

### 2.2. Measures

A comprehensive, self-administered, anonymous questionnaire was used for data collection. The questionnaire consisted of twenty-three questions divided into four main sections. The first section comprised seven items that explored the sociodemographic (age, gender, marital status, place of family medicine practice, education, length of service) and employment (type of practice) characteristics of the GPs. The second section included five items that explored the exposure of the GPs from both surveyed countries to COVID-19 as well as the education of the study participants regarding the preventative measures of the coronavirus infection. The third section comprised seven items that explored the attitudes of the GPs toward COVID-19 prevention and control and the influence of the pandemic on healthcare workers. The fourth section included four items regarding the COVID-19 prevention and control practices of the GPs during the restrictive and non-restrictive epidemiological measures in each country. It took around 20 min for the participants to complete the questionnaire. The questionnaire used in this study was constructed by using some of the questions from similar questionnaires used by Moodley et al. and Mohammed Basheeruddin Asdaq et al. in their studies, which were reformulated according to Croatian and Bosnian language expressions [22,23]. The attitude items used a five-point Likert response scale (strongly agree; agree; neither agree, neither disagree; disagree; strongly disagree). The practice items measured both individual and facility practices according to the World Health Organization guidelines that were used as the foundation for most of the items [27]. The questionnaire used in this study was validated on a small group of GPs from eastern Croatia (n = 30) during the year 2021; minor changes were made at that time to improve the readability and clarity of the questionnaire. The final attitude and practice scales in the questionnaire had an acceptable internal reliability with Cronbach’s alpha values of 0.519 and 0.533, respectively.

### 2.3. Statistical Analysis

The normality of the data distribution was tested with the Kolmogorov–Smirnov test, and thereafter all the data were processed by methods of descriptive statistics. All the categorical variables were expressed in absolute and relative frequencies, while the numerical variables were expressed as median and interquartile ranges. The χ^2^-test and Fisher’s exact test were used for the comparison of the categorical variables between the groups, while the Mann–Whitney U test and the Kruskal–Wallis test were used for the comparison of the numerical variables between the groups. In all the statistical analyses, two-sided *p*-values of 0.05 were considered significant. The level of statistical significance was set at *p* < 0.05. Statistical analysis was performed using the IBM SPSS statistical package, version 22.0 (SPSS Inc., Chicago, IL, USA).

## 3. Results

### 3.1. Study Participants

The study included 200 GPs from Croatia and the Federation of Bosnia and Herzegovina, with 100 GPs from each country. The median age of the participants was 42.00 years (interquartile range 31.00–57.75), and 74.5% were females. A total of 55.0% of the participants were in the younger age group (45 years or less), and 45.0% of the participants were in the older age group (46 years or more). Being single was reported by 36.0% of the participants, while 64.0% were in a relationship. According to the place of their family medicine practice, 45.0% of the participants had their practice in the county or cantonal center, 43.5% of the participants had their practice in some other city in the county or canton, and 11.5% of the participants had their practice in a village or suburban settlement. Regarding education, 55.5% of GPs had a license for independent practice without finished specialization, and 44.5% were specialists in family medicine. The median length of service was 12.00 years (interquartile range 3.00–28.00), with 52.0% of the participants with a length of service from 0 to 14 years and 48.0% of the participants with a length of service of 15 years or more. Regarding the type of family medicine practice, 75.0% of the participants were employees of a health center, while 25.0% of them had a concession or a private practice. The sociodemographic and employment characteristics of the GPs from Croatia and Bosnia and Herzegovina are presented in Table 1.

Regarding the GPs’ coronavirus exposure or infection, 70.0% of the participants had had COVID-19 or had lived in the same household with a family member who had COVID-19. Regarding the education about the preventative measures of coronavirus infection, 44.5% of the GPs reported finishing formal education on the prevention of infectious diseases and occupational safety, 39.0% of the GPs reported finishing formal education on adequate hand hygiene, 96.5% of the GPs reported having an official protocol regarding protection from COVID-19 in family medicine in their place of work, and 40.0% of the GPs reported finishing formal education on COVID-19 prevention for general practitioners. The results regarding coronavirus exposure or infection and education regarding the preventative measures of coronavirus infection among the GPs are presented in Table 2.

### 3.2. Attitudes toward COVID-19 Prevention and Control and the Influence of the Pandemic on Healthcare Workers

The median number of positive attitudes toward COVID-19 prevention and control was 5.00 with an interquartile range between 4.00 and 6.00. The study revealed that there was a significant difference in the overall number of positive attitudes toward COVID-19 prevention and control between the GPs from Croatia and Bosnia and Herzegovina (Mann–Whitney’s U test; *p* = 0.014), where the GPs from Croatia displayed a larger number of positive attitudes toward COVID-19 prevention and control and the influence of the pandemic on healthcare workers. The 9ndividual attitudes of the GPs from Croatia and Bosnia and Herzegovina toward COVID-19 prevention and control and the influence of the pandemic on healthcare workers are presented in Table 3.

The study revealed that different factors were associated with the established attitudes of the GPs from Croatia and Bosnia and Herzegovina toward COVID-19 prevention and control, and these results are presented in Table 4. In the group of Croatian GPs, it was established that the GPs who had finished formal education on the prevention of infectious diseases and occupational safety had more positive attitudes toward COVID-19 prevention and control (*p* = 0.019). Among the Bosnian GPs, it was determined that there were more positive attitudes toward COVID-19 prevention and control among the older GPs (46 years or more) (*p* = 0.007), males (*p* = 0.027), GPs with a longer length of service (15 or more years) (*p* = 0.006), GPs who had finished formal education on the prevention of infectious diseases and occupational safety (*p* < 0.001), GPs who had finished formal education on adequate hand hygiene (*p* < 0.001), and GPs who had finished formal education on COVID-19 prevention for GPs (*p* = 0.002).

### 3.3. Practices Regarding COVID-19 Prevention and Control

The median number of positive practices regarding COVID-19 prevention and control was 4.00 with an interquartile range between 3.00 and 4.00. The study revealed that there was no significant difference in the overall number of positive practices regarding COVID-19 prevention and control between the GPs from Croatia and Bosnia and Herzegovina (Mann-Whitney’s U test; *p* = 0.077).

The COVID-19 prevention and control practices of the GPs from Croatia and Bosnia and Herzegovina during the restrictive and non-restrictive epidemiological measures in each country are presented in Table 5.

The study showed that different factors were associated with the established practices of the GPs from Croatia and Bosnia and Herzegovina regarding COVID-19 prevention and control, and these results are presented in Table 6. The study revealed that among the Croatian GPs, more positive practices regarding COVID-19 prevention and control were reported by older GPs (46 years or more) (*p* = 0.009), females (*p* = 0.003), GPs who had a partner (*p* = 0.022), GPs who were specialists in family medicine (*p* = 0.015), GPs with a longer length of service (15 or more years) (*p* = 0.007), and GPs who had finished formal education on the prevention of infectious diseases and occupational safety (*p* = 0.046). The study further discovered that among the Bosnian and Herzegovinian GPs, none of the investigated factors was associated with established practices regarding COVID-19 prevention and control.

## 4. Discussion

The present study assessed the attitudes and practices of GPs from Croatia and Bosnia and Herzegovina regarding COVID-19 prevention and control and the factors influencing the observed attitudes and practices in the surveyed population. This is a highly important issue since primary healthcare workers have a significant role in the containment of the spread of COVID-19 in any particular country, and it has been proven that a response built around primary care is also a more cost-effective measure, which is highly important for all healthcare systems in today’s modern world [6]. To better understand the practices of primary healthcare workers regarding COVID-19 in a particular country, it is very important to know their attitudes toward the same issue as well as the various factors that have a strong influence on such attitudes, because it has been shown that participants with higher positive attitude scores are also more likely to score higher in their practice [21,22,23,28,29,30,31,32]. The connection between attitudes and practices can be explained by the reasoned action theory, which states that a person’s intention to undertake a specific behavior is a function of their attitude towards that behavior [33].

The present study revealed that the majority of the studied population had satisfactory attitudes toward different aspects of COVID-19 prevention and control as well as toward the influence of the pandemic on healthcare workers, which was in concordance with some other similar studies [7,15,19,22,23,34,35,36], although there is also research in which only one-third of healthcare workers in primary health care centers showed satisfactory attitudes toward investigated issue [31]. One possible explanation for the discrepancies between the results of a study conducted by Albahri et al. and other studies, including ours, could be the time point of the pandemic when the research was carried out. It is possible that at the beginning of the pandemic (the year 2020), due to a lot of unknown aspects concerning the new virus, the attitudes of healthcare workers toward its prevention and control were somewhat less positive than in a later time of the pandemic (the years 2021 and 2022).

This study further discovered that there were some significant differences between the surveyed countries regarding the attitudes of GPs toward COVID-19 prevention and control. Considering the overall number of positive attitudes, it was established that the GPs from Croatia displayed a larger number of positive attitudes toward COVID-19 prevention and control and toward the influence of the pandemic on healthcare workers in comparison to their colleagues from Bosnia and Herzegovina. Similarly, the study that included GPs from several different countries, where the majority of them were from Turkey, Greece, and the United States, also showed that GPs from the United States had more positive attitudes toward COVID-19 in comparison to their colleagues from Greece and Turkey [7]. One possible explanation for the observed differences in the GPs’ attitudes in the cited study as well as in our study is the cultural differences among the countries that strongly influence the COVID-19 risk perception in the surveyed countries and the attitudes of GPs toward the different aspects of COVID-19 prevention and control [37].

When looking separately at the individual attitudes, the present study showed that the GPs from Croatia better understood that the usage of personal protective equipment (PPE) prevents COVID-19 infection in patients in comparison to the GPs from Bosnia and Herzegovina, while both groups of GPs were equally aware that the usage of PPE prevents COVID-19 infection among healthcare workers. Both groups of study participants thought that GPs should treat COVID-19 patients, and they all equally considered that GPs were sufficiently educated for their work during the COVID-19 pandemic and that the GPs’ offices were sufficiently equipped with protective equipment for work during the COVID-19 pandemic. Similar studies conducted elsewhere also showed that the majority of surveyed healthcare workers would wear the required PPE and would treat COVID-19 patients [7,22]. A study in Saudi Arabia showed that the majority of physicians believed that they were sufficiently educated for their work during the COVID-19 pandemic [23], but a study conducted in Sierra Leone showed that healthcare workers thought that their health facilities were not sufficiently equipped for an adequate response to the COVID-19 outbreak [38]. Croatian GPs were more aware that the COVID-19 pandemic increased the level of stress among GPs, but at the same time they were more convinced that their country was successful in the containment of the spread of COVID-19 in comparison to the GPs from Bosnia and Herzegovina. Studies conducted in Saudi Arabia, Sierra Leone, and Nigeria also showed that the majority of healthcare workers believed that their countries would contain the COVID-19 pandemic successfully [23,34,38,39].

The determined differences among the surveyed countries, besides their probable cultural differences, can also be attributed to the different sociodemographic and employment characteristics of the surveyed GPs, as well as to the overall organization of the healthcare systems in Croatia and Bosnia and Herzegovina. Namely, this study revealed that among the Croatian GPs there were no significant associations between the various sociodemographic and employment variables and the GPs attitudes, and the only factor that was significantly associated with more positive attitudes toward the COVID-19 prevention and control was whether the GPs had finished formal education on the prevention of infectious diseases and occupational safety. Studies from several different countries also did not find significant connections between sociodemographic and employment variables and the attitudes of healthcare workers [7,20,23,29,30,40,41]. Studies from Saudi Arabia, China, and Pakistan confirmed that the participation of healthcare workers in formal education on the prevention of infectious diseases and occupational safety contributed significantly to their positive attitudes toward COVID-19 prevention and control, and this was also proven in this study, both for the Croatian and Bosnian GPs [28,30,34,42].

Considering the Bosnian GPs, the present study further discovered that more positive attitudes toward COVID-19 prevention and control were also found in older GPs (46 years or more), males, GPs with a longer length of service (15 or more years), GPs who had finished formal education on COVID-19 prevention for GPs, and GPs who had finished formal education on adequate hand hygiene. Studies in India also showed that older healthcare workers had more positive attitudes toward COVID-19 prevention and control [19,21], while studies from Saudi Arabia and Greece, as well as the results of this study concerning the Croatian GPs, did not find a connection between age and GPs attitudes toward COVID-19 prevention and control [29,34]. A study from Nigeria, however, discovered that older healthcare workers had more negative attitudes toward COVID-19 prevention and control [39]. While studies from Saudi Arabia, Sierra Leone, and Jordan also discovered that male healthcare workers, like the male GPs from Bosnia and Herzegovina in this study, had more positive attitudes toward COVID-19 prevention and control [15,32,38], another study from Saudi Arabia, and studies from India and China, contrary to the results among the Bosnian GPs in this study, showed that females had more positive attitudes than males [19,34,43]. Finally, a study that included GPs from the United States, Turkey, and Greece and another study that included only GPs from Greece found no connection between gender and attitudes toward COVID-19 prevention and control [7,29]. A study among frontline healthcare workers in India also discovered that healthcare workers with a longer length of service had more positive attitudes toward COVID-19 prevention and control [21]. Unlike the results concerning the Bosnian GPs in this study, a study conducted by Gokdemir et al. did not find a connection between the length of service and attitudes toward COVID-19 prevention and control [7], while studies from Nigeria and Sierra Leone showed that healthcare workers with longer lengths of service had more negative attitudes toward COVID-19 prevention and control [38,39]. Like our study results for the GPs from Bosnia and Herzegovina, a study conducted among frontline healthcare workers in Nepal also showed that more positive attitudes toward COVID-19 prevention and control were connected with having finished additional formal education regarding COVID-19 prevention [44]. In addition, studies among healthcare workers in Saudi Arabia and Jordan showed that those who participated in infection control training were more likely to have positive attitudes toward COVID-19 prevention and control [32,34].

According to the Center for Diseases Control and Prevention, handwashing with soap and water for at least 20 s or the use of alcohol-based hand sanitizers when soap and water are not available is the first line of defense in stopping the spread of COVID-19, and because of this, it is not surprising that the Bosnian GPs from this study who had finished formal education on adequate hand hygiene had, at the same time, more positive attitudes toward COVID-19 prevention and control [45]. This finding confirms one more time that there is a strong correlation between GPs’ knowledge about a particular issue and their attitudes toward the same issue, and that additional education on a particular issue can facilitate positive changes in physicians’ attitudes toward the same issue [17].

Considering the practices of the Croatian and Bosnian GPs regarding COVID-19 prevention and control, the present study showed that the GPs from both countries displayed a strong adherence to adequate practices regarding COVID-19 prevention and control, which was similar to the results of several studies conducted elsewhere [19,21,29,30,31,34,35,36,38,44]. Unlike for the attitudes, this study further revealed that there was no significant difference in GPs’ practices regarding COVID-19 prevention and control between the the study participants from Croatia and Bosnia and Herzegovina. However, when looking separately into the individual practices, this study clearly showed that there was a significant difference between the surveyed countries regarding the usage of protective facial masks in everyday life during the non-restrictive epidemiological measures in a particular country, where the Croatian GPs significantly more often practiced the described preventive behavior in comparison to their colleagues in Bosnia and Herzegovina. The discovered differences could be associated with the different social and cultural norms that exist in these neighboring countries, because it has been proven that practices of wearing or not wearing facial masks are strongly influenced by several factors such as personal interpretations of solidarity and responsibility, individual perceptions of COVID-19 risk, and cultural and societal traditions [46]. Furthermore, a study conducted in the general population of Bosnia and Herzegovina during the restrictive epidemiological measures in the country showed that, despite high fines, over 13% of citizens did not wear facial masks, showing that the whole society of the country probably had a different pattern of COVID-19 practices from the beginning of the outbreak in comparison to that of Croatia [47].

In addition to this, this study also showed different patterns of association between more positive practices regarding COVID-19 prevention and control and the different sociodemographic and employment characteristics of the study participants from the surveyed countries. It was established that among the Bosnian GPs, there were no significant associations between the various sociodemographic and employment variables and the GPs’ practices, while among the Croatian GPs, there were several factors that were significantly associated with more positive practices of the Croatian GPs regarding COVID-19 prevention and control.

It was determined that among the Croatian GPs, more positive practices regarding COVID-19 prevention and control were reported by older GPs (46 years or more), females, GPs who had a partner, GPs who were specialists in family medicine, GPs with a longer length of service (15 or more years), and GPs who had finished formal education on the prevention of infectious diseases and occupational safety. Studies conducted in Greece and Dubai also did not find associations between various sociodemographic and employment variables and GPs’ practices [29,31]. However, some studies showed significant correlations between particular sociodemographic and employment variables and GPs’ practices regarding COVID-19 prevention and control. Considering age, a study conducted in India showed that, like the Croatian GPs in this study, older healthcare workers from this country more frequently displayed positive practices regarding COVID-19 prevention and control in comparison to their younger colleagues [19]. Furthermore, like the Croatian GPs in this study, a study from China showed that female GPs expressed more positive practices regarding COVID-19 prevention and control [43], while a study in Saudi Arabia discovered that male GPs from Saudi Arabia were more likely to comply with appropriate practices regarding COVID-19 prevention and control in comparison to their female colleagues [34]. Unlike the Croatian GPs in this study, studies in Riyadh, Saudi Arabia, and India revealed that single healthcare workers more frequently displayed positive practices regarding COVID-19 prevention and control in comparison to their married colleagues [19,23]. Opposite to the results of this study for the Croatian GPs, a study in Greece did not reveal a significant difference in the displayed practices regarding COVID-19 prevention and control between specialists in family medicine and doctors without specialization [29]. Regarding the length of service, studies in Pakistan and India showed that, like the Croatian GPs in this study, healthcare workers with a longer length of service reported more positive practices regarding COVID-19 prevention and control [21,30]. Like the Croatian GPs in this study, studies from Saudi Arabia and Jordan showed that healthcare workers who had finished formal education on the prevention of infectious diseases and occupational safety displayed more positive practices regarding COVID-19 prevention and control [32,34]. Additionally, a study among frontline healthcare workers in Nepal showed that more positive practices regarding COVID-19 prevention and control were connected with having finished additional formal education regarding COVID-19 prevention [44].

To the best of our knowledge, this was the first study that analyzed the attitudes and practices of GPs from Croatia and Bosnia and Herzegovina, thus giving an in-depth insight and fulfilling the knowledge gap regarding the important issue of primary healthcare workers’ involvement in resolving the COVID-19 outbreak in this part of Europe. The present study included GPs working in state institutions as well as those who were working in private practices, which is highly important because a similar study conducted elsewhere stated that healthcare workers working in the private sector may have different attitudes regarding the pandemic [21].

Despite the aforementioned important strengths, this study is not without limitations. The present study was cross-sectional, and because of the employed study design, the demonstration of any causality between the outcome and explanatory variables is limited. The research tool was self-administered and dependent on the honesty and accuracy of the participant’s answers, and because of this, the risk of social desirability of disclosed attitudes and practices cannot be completely ignored. The study in Bosnia and Herzegovina was limited to only one entity, i.e., the Federation of Bosnia and Herzegovina, and the results from this country might be somewhat different if GPs from other entities, i.e., the Republika Srpska and the Brčko District, were also included in the study. The final sample size of this study was rather small, although the response rate was acceptable, which was a consequence of the quite large portion of discarded questionnaires that were incomplete. The majority of those incomplete questionnaires came from Croatian GPs and were probably connected with the enormous workload of Croatian GPs during the COVID-19 pandemic because it is well-known fact that even before the pandemic, Croatian GPs were facing an increased workload [48].

Despite all the previously mentioned limitations, this study revealed important associations between GPs’ attitudes and practices regarding COVID-19 prevention and control and pointed to the possible factors that may have influenced the investigated variables and can potentially explain the differences between the surveyed neighboring European countries. Furthermore, the present study raised some new questions concerning the investigated issues, namely, a recent study by Gokdemir et al. [7] showed that the personality traits of healthcare workers could strongly influence their attitudes toward COVID-19 prevention and control. Thus, there is a need for new studies of Croatian and Bosnian GPs’ attitudes and practices regarding COVID-19 prevention and control with a larger sample size in both countries, where in the sample from Bosnia and Herzegovina, the GPs from the whole territory of Bosnia and Herzegovina should be included, and the survey should be conducted with a questionnaire that comprises additional items regarding the personality traits of the study participants.

## 5. Conclusions

The present study revealed that the attitudes and practices of GPs from Croatia and Bosnia and Herzegovina regarding COVID-19 prevention and control were satisfactory. The study further showed that the Croatian GPs displayed a larger number of positive attitudes toward COVID-19 prevention and control and the influence of the pandemic on healthcare workers in comparison to their colleagues from Bosnia and Herzegovina, while there was no significant difference in the practices regarding COVID-19 prevention and control among the surveyed countries. Finally, the study disclosed that the different sociodemographic and employment characteristics of the study participants strongly influenced their attitudes and practices regarding the prevention and control of COVID-19, whereas the cultural differences between Croatia and Bosnia and Herzegovina, as well as the organizational specificities of their healthcare systems, probably modified the observed differences in the individual patterns of associations between the outcome and explanatory variables in the surveyed neighboring countries.

## Figures and Tables

**Table 1:** Sociodemographic and employment characteristics of GPs from Croatia and Bosnia and Herzegovina.

Characteristics	Croatia N (%)	Bosnia and Herzegovina N (%)	*p* *
**Age group (years)**			
Younger (45 years or less)	51 (51.0)	59 (59.0)	0.256
Older (46 years or more)	49 (49.0)	41 (41.0)	
**Gender**			
Male	18 (18.0)	33 (33.0)	0.023
Female	82 (82.0)	67 (67.0)	
**Marital status**			
With a partner	55 (55.0)	73 (73.0)	0.012
Single	45 (45.0)	27 (27.0)	
**Place of family medicine practice**			
County or cantonal center	50 (50.0)	40 (40.0)	<0.001
Other cities in the county or canton	31 (31.0)	56 (56.0)	
Villages or suburban settlements	19 (19.0)	4 (4.0)	
**Education**			
Doctors with a license for independent practice without finished specialization	57 (57.0)	54 (54.0)	0.776
Specialist in family medicine	43 (43.0)	46 (46.0)	
**Length of service (years)**			
0–14 years	48 (48.0)	56 (56.0)	0.258
15 or more years	52 (52.0)	44 (44.0)	
**Type of practice**			
An employee of a health center	50 (50.0)	100 (100.0)	<0.001
Concession or a private practice	50 (50.0)	0	

* χ^2^-test.

**Table 2:** Characteristics of GPs from Croatia and Bosnia and Herzegovina according to coronavirus exposure or infection and according to their education regarding the preventative measures of coronavirus infection.

Characteristics	Croatia N (%)	Bosnia and Herzegovina N (%)	*p*
**The GP or his/her family member living in the same household had COVID-19.**			
Yes	57 (57.0)	83 (83.0)	<0.001 *
No	43 (43.0)	17 (17.0)	
**The GP finished formal education on the prevention of infectious diseases and occupational safety.**			
Yes	46 (46.0)	43 (43.0)	0.776 *
No	54 (54.0)	57 (57.0)	
**The GP finished formal education on adequate hand hygiene.**			
Yes	37 (37.0)	41 (41.0)	0.664 *
No	63 (63.0)	59 (59.0)	
**In the GP’s place of work, there was an official protocol regarding protection from COVID-19 in family medicine.**			
Yes	99 (99.0)	94 (94.0)	0.118 ^†^
No	1 (1.0)	6 (6.0)	
**The GP finished formal education on COVID-19 prevention for GPs.**			
Yes	42 (42.0)	38 (38.0)	0.665 *
No	58 (58.0)	62 (62.0)	

* χ^2^-test; ^†^ Fisher’s exact test; GP—general practitioner.

**Table 3:** The attitudes of GPs from Croatia and Bosnia and Herzegovina toward COVID-19 prevention and control and the influence of the pandemic on healthcare workers.

Statements	Strongly Agree N (%)	Agree N (%)	Neither Agree nor Disagree N (%)	Disagree N (%)	Strongly Disagree N (%)	*p*
**The usage of PPE prevents COVID-19 among healthcare workers.**						
Croatian GPs	30 (30.0)	57 (57.0)	10 (10.0)	1 (1.0)	2 (2.0)	0.218 ^†^
Bosnian GPs	22 (22.0)	55 (55.0)	12 (12.0)	4 (4.0)	7 (7.0)	
**The usage of PPE prevents COVID-19 in patients.**						
Croatian GPs	29 (29.0)	58 (58.0)	9 (9.0)	3 (3.0)	1 (1.0)	<0.001 ^†^
Bosnian GPs	18 (18.0)	23 (23.0)	27 (27.0)	28 (28.0)	4 (4.0)	
**GPs should treat COVID-19 patients.**						
Croatian GPs	42 (42.0)	41 (41.0)	9 (9.0)	7 (7.0)	1 (1.0)	0.202 ^†^
Bosnian GPs	43 (43.0)	50 (50.0)	3 (3.0)	4 (4.0)	0	
**GPs are sufficiently educated for their work during the COVID-19 pandemic.**						
Croatian GPs	13 (13.0)	46 (46.0)	23 (23.0)	16 (16.0)	2 (2.0)	0.235 ^†^
Bosnian GPs	22 (22.0)	38 (38.0)	18 (18.0)	16 (16.0)	6 (6.0)	
**GPs’ offices are sufficiently equipped with protective equipment for work during the COVID-19 pandemic.**						
Croatian GPs	5 (5.0)	31 (31.0)	27 (27.0)	26 (26.0)	11 (11.0)	0.163 *
Bosnian GPs	8 (8.0)	41 (41.0)	15 (15.0)	29 (29.0)	7 (7.0)	
**The COVID-19 pandemic will increase the level of stress among GPs.**						
Croatian GPs	66 (66.0)	31 (31.0)	3 (3.0)	0	0	0.006 ^†^
Bosnian GPs	46 (46.0)	47 (47.0)	2 (2.0)	4 (4.0)	1 (1.0)	
**My country is successful in the containment of the spread of COVID-19.**						
Croatian GPs	3 (3.0)	46 (46.0)	34 (34.0)	14 (14.0)	3 (3.0)	0.005 ^†^
Bosnian GPs	1 (1.0)	34 (34.0)	26 (26.0)	36 (36.0)	3 (3.0)	

* χ^2^-test; ^†^ Fisher’s exact test; PPE—personal protective equipment; Croatian GPs—GPs from Croatia; Bosnian GPs—GPs from Bosnia and Herzegovina.

**Table 4:** Associations between attitudes of GPs from Croatia and Bosnia and Herzegovina toward COVID-19 prevention and control and their sociodemographic and employment characteristics, exposure to COVID-19, and education regarding the preventative measures of coronavirus infection.

Factors	Croatian GPs		Bosnian GPs	
	Mean (Standard Deviation)	*p*	Mean (Standard Deviation)	*p*
**Sociodemographic and** **employment characteristics**				
**Age group**				
Younger (45 years or less)	4.84 (1.362)	0.313 *	4.20 (1.243)	0.007 *
Older (46 years or more)	5.12 (1.438)		4.88 (1.487)	
**Gender**				
Male	5.33 (1.138)	0.251 *	4.91 (1.355)	0.027 *
Female	4.90 (1.445)		4.27 (1.355)	
**Marital status**				
With a partner	4.80 (1.458)	0.155 *	4.62 (1.440)	0.086 *
Single	5.20 (1.307)		4.11 (1.155)	
**Place of family medicine practice**				
County or cantonal center	5.02 (1.270)	0.441 ^†^	4.68 (1.309)	0.451 ^†^
Other cities in the county or canton	4.71 (1.716)		4.38 (1.459)	
Villages or suburban settlements	5.32 (1.108)		4.00 (0.816)	
**Education**				
Doctors with a license for independent practice without having finished specialization	4.96 (1.426)	0.915 *	4.26 (1.306)	0.060 *
Specialist in family medicine	5.00 (1.380)		4.74 (1.437)	
**Length of service**				
0–14 years	4.83 (1.389)	0.318 *	4.18 (1.281)	0.006 *
15 or more years	5.12 (1.409)		4.86 (1.424)	
**Type of practice**				
An employee of a health center	5.22 (1.314)	0.098 *	4.48 (1.382)	NA
Concession or a private practice	4.74 (1.454)		0	
**The exposure of GPs to COVID-19** **and their education regarding the preventative measures of coronavirus infection**				
**The GP or his/her family member living in the same household had COVID-19**				
Yes	4.88 (1.324)	0.307 *	4.58 (1.354)	0.101 *
No	5.12 (1.499)		4.00 (1.458)	
**The GP finished formal education on the prevention of infectious diseases and occupational safety**				
Yes	5.33 (1.248)	0.019 *	5.16 (1.233)	<0.001 *
No	4.69 (1.464)		3.96 (1.267)	
**The GP finished formal education on adequate hand hygiene**				
Yes	4.92 (1.256)	0.594 *	5.15 (1.085)	<0.001 *
No	5.02 (1.486)		4.02 (1.383)	
**In the GP’s place of work, there was an official protocol regarding protection from COVID-19 in family medicine**				
Yes	4.96 (1.392)	0.142 *	4.50 (1.318)	0.830 *
No	7.00 (0.000)		4.17 (2.317)	
**The GP finished formal education on COVID-19 prevention for GPs**				
Yes	5.02 (1.316)	0.844 *	5.00 (1.273)	0.002 *
No	4.95 (1.468)		4.16 (1.357)	

* Mann–Whitney’s U test; ^†^ Kruskal–Wallis’s test; NA—not applicable; Croatian GPs—GPs from Croatia; Bosnian GPs—GPs from Bosnia and Herzegovina; GP—general practitioner.

**Table 5:** The COVID-19 prevention and control practices of GPs from Croatia and Bosnia and Herzegovina during the restrictive and non-restrictive epidemiological measures in each country.

Practice	Croatia N (%)	Bosnia and Herzegovina N (%)	*p*
**During the restrictive epidemiological measures in my country,** **I always wore a protective facial mask when I needed to do some things outside of my home or my office (for example shopping for groceries).**			
Yes	98 (98.0)	95 (95.0)	0.445 ^†^
No	2 (2.0)	5 (5.0)	
**During the restrictive epidemiological measures in my country,** **I always used all available PPE during working with patients in my office.**			
Yes	84 (84.0)	84 (84.0)	>0.999 *
No	16 (16.0)	16 (16.0)	
**During the non-restrictive epidemiological measures in my country,** **I always wore a protective facial mask when I needed to do some things outside of my home or my office (for example shopping for groceries).**			
Yes	97 (97.0)	77 (77.0)	<0.001 *
No	3 (3.0)	23 (23.0)	
**During the non-restrictive epidemiological measures in my country,** **I always used all available PPE during working with patients in my office.**			
Yes	72 (72.0)	71 (71.0)	>0.999 *
No	28 (28.0)	29 (29.0)	

* χ^2^-test; ^†^ Fisher’s exact test; PPE—personal protective equipment.

**Table 6:** Associations between the COVID-19 prevention and control practices of GPs from Croatia and Bosnia and Herzegovina and their sociodemographic and employment characteristics, exposure to COVID-19, and their education regarding the preventative measures of coronavirus infection.

Factors	Croatian GPs		Bosnian GPs	
	Mean (Standard Deviation)	*p*	Mean (Standard Deviation)	*p*
**Sociodemographic and** **employment characteristics**				
**Age group**				
Younger (45 years or less)	3.31 (0.883)	0.009 *	3.17 (1.003)	0.138 *
Older (46 years or more)	3.71 (0.612)		3.41 (0.974)	
**Gender**				
Male	3.00 (0.970)	0.003 *	3.27 (0.977)	0.865 *
Female	3.62 (0.696)		3.27 (1.009)	
**Marital status**				
With a partner	3.71 (0.533)	0.022 *	3.27 (1.031)	0.721 *
Single	3.27 (0.963)		3.26 (0.903)	
**Place of family medicine practice**				
County or cantonal center	3.58 (0.673)	0.864 ^†^	3.33 (0.971)	0.653 ^†^
Other cities in the county or canton	3.45 (0.888)		3.29 (0.948)	
Villages or suburban settlements	3.42 (0.902)		2.50 (1.732)	
**Education**				
Doctors with a license for independent practice without having finished specialization	3.33 (0.893)	0.015 *	3.19 (1.029)	0.348 *
Specialist in family medicine	3.74 (0.539)		3.37 (0.951)	
**Length of service**				
0–14 years	3.29 (0.898)	0.007 *	3.09 (1.100)	0.055 *
15 or more years	3.71 (0.605)		3.50 (0.792)	
**Type of practice**				
An employee of a health center	3.36 (0.875)	0.055 *	3.27 (0.993)	NA
Concession or a private practice	3.66 (0.658)		0	
**The exposure of GPs to COVID-19 and their education regarding the coronavirus infection preventative measures**				
**The GP or his/her family member living in the same household had COVID-19**				
Yes	3.47 (0.826)	0.630 *	3.27 (1.001)	0.883 *
No	3.56 (0.734)		3.29 (0.985)	
**The GP finished formal education on the prevention of infectious diseases and occupational safety**				
Yes	3.70 (0.591)	0.046 *	3.49 (0.703)	0.182 *
No	3.35 (0.894)		3.11 (1.145)	
**The GP finished formal education on adequate hand hygiene**				
Yes	3.49 (0.804)	0.777 *	3.56 (0.550)	0.095 *
No	3.52 (0.780)		3.07 (1.172)	
**In the GP’s place of work, there was an official protocol regarding protection from COVID-19 in family medicine**				
Yes	3.52 (0.787)	0.269 *	3.24 (1.013)	0.385 *
No	3.00 (0.000)		3.67 (0.516)	
**The GP finished formal education on COVID-19 prevention for GPs**				
Yes	3.60 (0.767)	0.255 *	3.29 (0.984)	0.987 *
No	3.45 (0.799)		3.26 (1.007)	

* Mann–Whitney’s U test; ^†^ Kruskal–Wallis’s test; NA—not applicable; Croatian GPs—GPs from Croatia; Bosnian GPs—GPs from Bosnia and Herzegovina; GP—general practitioner.

## Data Availability

The data presented in this study are available on request from the corresponding authors.
